# Evaluating the Effectiveness of Patient-Centered Standardized Prophylaxis Processes in Enhancing Patient Satisfaction and Return Intentions

**DOI:** 10.3390/bs15010024

**Published:** 2024-12-30

**Authors:** Wen Tao, Tingfang Liu

**Affiliations:** School of Health Policy and Management, Chinese Academy of Medical Sciences & Peking Union Medical College, Beijing 100730, China; taowen@student.pumc.edu.cn

**Keywords:** dental prophylaxis, teeth cleaning, health service quality, standard process, patient centered, patient satisfaction, long-term health management, information system, patient return intention

## Abstract

This study aimed to evaluate the effectiveness of a patient-centered standardized prophylaxis process in improving patient satisfaction and intentions to return to dental clinics. Conducted in a first-tier city in China from 9 June to 26 July 2023, the cross-sectional survey included 826 patients from 38 dental clinics. Among the respondents, 438 received standardized prophylaxis services, while 388 were in the non-standardized group, with a mean age of 38.2 ± 12.1 years and 50.24% male participation. The results revealed that patients in the standardized prophylaxis group reported significantly higher satisfaction scores (average 4.74 vs. 3.34, *p* < 0.0001) and greater intentions to return (average 4.77 vs. 4.10, *p* < 0.0001) compared to those receiving non-standardized care. The ordinal logistic regression analysis identified patient satisfaction as a strong predictor of return intention, with an odds ratio of 24.487, while the standardized service group had an odds ratio of 16.063, indicating a substantial positive effect on satisfaction. Age was also found to significantly affect return intention, reflected in an odds ratio of 0.969. Furthermore, the model showed strong predictive accuracy, which was supported by a Somers’ D value of 0.806. Additionally, an impressive 89.9% of the observations were correctly ordered, which strengthens the reliability of the findings. These outcomes highlight the significant contribution of patient-centered, standardized practices to the satisfaction of the patients as well as the development of their desire to return for follow-up care. However, the findings should be interpreted with caution due to the study’s cross-sectional nature, which limits causal inferences, and the specific demographic characteristics that may affect the general applicability of the results. Further research is needed to explore these dynamics across diverse populations and settings.

## 1. Introduction

Oral health is of key importance to good health (Barranca-Enríquez & Romo-González, 2022). It includes strategies for the prevention and management of oral diseases (Niranjan et al., 2017) and the maintenance of optimal oral function (Sedghi et al., 2021). Moreover, it has been recognized as a major public health issue (Peres et al., 2019), causing enormous harm to physical health (Hescot, 2017) and social interactions (Donnelly et al., 2016) as well as the overall quality of life (Spanemberg et al., 2019).

In China, dental clinics have been the main providers of oral health care (Liu et al., 2016). Dental prophylaxis is considered the most critical element of oral health advancement (Newton & Asimakopoulou, 2017). It is also undeniably the most important aspect of building a trustworthy relationship between doctors and patients (Sbaraini et al., 2012). The quality of dental prophylaxis services is, hence, the main factor (Naseem et al., 2017). As for this, it not only links to oral health in a short term perspective or even results in better patient continuity (Wiegand & Schlueter, 2014) but it is also a platform for further developments in terms of long-term patient participation and commitment to good dental practice (Martignon et al., 2019; Sbaraini et al., 2012).

Dental prophylaxis, as a basic procedure, is more than just teeth cleaning (Minihan-Anderson, 2023). It is an in-depth eternal process that fosters patient–practitioner relationships (Cristache et al., 2024) and also affecting patient satisfaction and revisit intentions (Khan, 2019). Several previous studies have evidenced that satisfaction and returning patients if the services were well delivered to them are valuable determinants in assessing the healthcare process quality (Al Ghanem et al., 2023).

However, the number of studies investigating the standardization of dental prophylaxis services in Chinese dental institutions is low, as reported in the article by Zhong (2019), in spite of the fact that the need for quality assurance in this sector has been increasing (Aldahmashi & Amaqa, 2022). The standardization of dental prophylaxis is especially important in the Chinese context (Ou et al., 2014). Data regarding oral health show a shocking prevalence of poor oral health (Zhang et al., 2022) and a heavy dependence on marketing rather than service quality (Chen, 2022), causing low patient revisit rates (Gao et al., 2020).

This study aims to address the following research questions: (1) How does the implementation of a patient-centered standardized prophylaxis process impact patient satisfaction and intentions to return in dental clinics in China? (2) What specific factors contribute to the success of standardized dental prophylaxis? Our hypothesis is that standardized dental prophylaxis services will significantly improve patient satisfaction and intentions to return compared to non-standardized services.

Our study will clarify the significance of patient-centered service experiments in dental clinics in China. We also want to objectively demonstrate the ability of customer feedback to set realistic service standards, therefore ensuring better patient outcomes. The quality of oral healthcare services is directly associated with patient satisfaction and return visits. These are the most competitive indicators of the success of these services. (Harris et al., 2011). Traditional approaches to service quality, like the SERVQUAL model, make use of the patients’ perceptions and expectations concerning the delivery of services. (Bentum-Micah et al., 2020). We are working with the college in collaboration with patients, clinic managers, and dentists to improve the quality of the dental treatment on offer through the means of implementing standardized dental prophylaxis processes. In order to highlight the way in which the two-dimensional techniques work, we did a survey in which dentists interact with the patients to solve initial problems. This was a rarely used method in China and our research article was the first to mention it (Ye et al., 2024). We have introduced a system plug-in that first automatically allots the self-inspection protocol for the dental practitioner during the appointment phase of the practice diagnosis. Finally, post-satisfaction surveys have been used to assess the revisit skills and satisfaction levels of patients.

Through this novel approach of incorporating patient feedback into service quality management, the project intends to provide real evidence and insights which will prove the successful application of standardized dental prophylaxis procedures within the Chinese dental clinic environment. We hope the findings from this research will contribute to the ongoing discourse on enhancing oral healthcare services and patient satisfaction of dental services in China.

## 2. Materials and Methods

### 2.1. Study Design and Setting

The study was conducted as a comparative and correlation analysis within private dental clinic settings in one of the China’s first-tier cities. We embedded a standardized prophylaxis service provider self-assessment system and a patient satisfaction assessment system within the clinic’s information system platform. The integrated system was strategically developed with the dual objectives of enhancing the caliber of dental prophylaxis services and laying the groundwork for the establishment of performance metrics and incentive systems for dental practitioners. Upon scheduling an appointment for prophylaxis, the system is automatically triggered, guiding practitioners through a process of self-assessment and simultaneously initiating a mechanism to collect patient feedback after the service has been rendered. This approach not only streamlines the evaluation process but also fosters a culture of continuous improvement by encouraging direct engagement with patient satisfaction. The study purpose was to evaluate patient satisfaction with the standardized dental prophylaxis service, the impact of standardization on satisfaction levels, and what factors really improve patient retention. The study encompassed a total of 38 dental clinics spots and covered a 1.5 month period.

**Figure 1:**
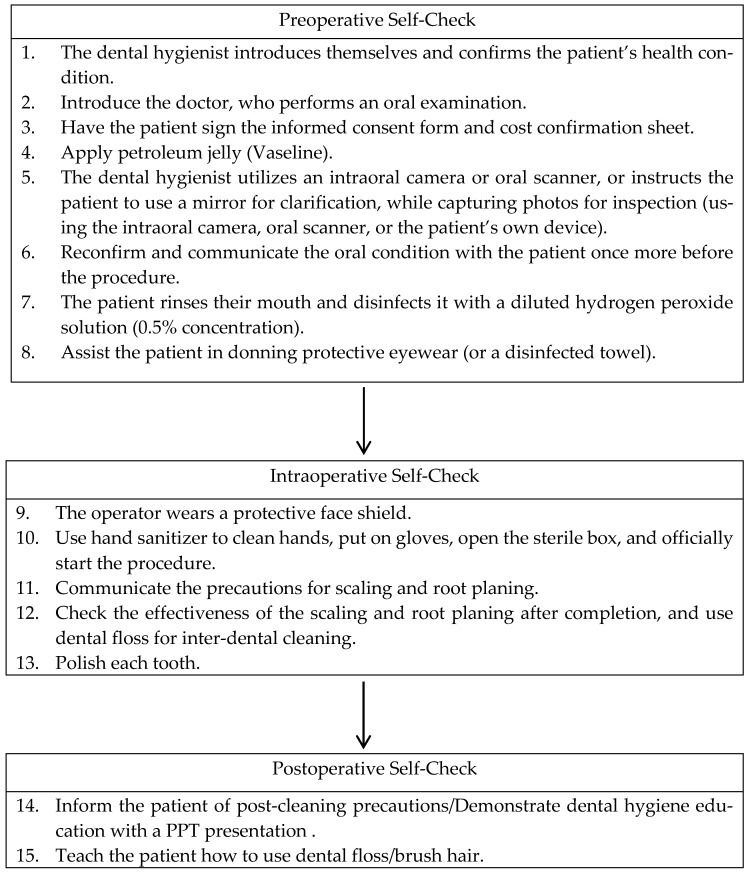
The patient-centered standardized prophylaxis processes steps.

In the domain of dental care, the patient-centered standardized prophylaxis processes have been meticulously designed to enhance patient engagement and satisfaction. These processes are underpinned by a collaborative approach that involves the patient, dental hygienist, dentist, and nurse in a comprehensive communication strategy aimed at improving the overall dental experience and outcomes. The dental practitioner would instruct and communicate with the patient in every step below. The check list is in Figure 1 as follows:

### 2.2. Participants and Study Procedures

After a two-week pilot phase dedicated to system testing and staff training in patient-centered standardized dental prophylaxis procedures, our intervention was launched across 38 dental clinics. From 9 June to 26 July 2023, we identified 8067 first-time customers. Out of these, 2260 participants engaged with the standardized prophylaxis service, filling out a self-assessment within the system. Among them, 438 participants also completed a newly designed patient satisfaction survey. In contrast, 5407 participants received non-standardized prophylaxis services during the same period, with 388 of these individuals completing a general satisfaction questionnaire for comparative analysis.

To ensure that participants fully understood the purpose of the study, we implemented a comprehensive informed consent procedure prior to data collection. Participants received a clear explanation of the study’s objectives, the procedures they would undergo, and the nature of their involvement. Additionally, they were informed that their responses were self-reported and were encouraged to ask questions for clarification at any time during the study. The participant selection process was carefully designed to ensure relevance and representativeness. We established specific criteria for inclusion, targeting first-time patients who sought dental prophylaxis services at the participating clinics. Recruitment methods included targeted advertising through social media platforms, local community outreach, and informational flyers distributed within the clinics. This multifaceted approach not only increased awareness of the study but also helped to reach a diverse pool of participants, thereby enhancing the generalizability of our findings. The study thus compared two groups: one that underwent standardized prophylaxis and another that did not, to evaluate the impact of standardization on patient satisfaction and return intention.

### 2.3. Measuring Tool

Employing a structured approach to engage participants (Boparai et al., 2018) and outline the study’s procedures, the satisfaction questionnaire was deliberately designed to assess two specific aspects of patient satisfaction within the framework of dental prophylaxis services: overall satisfaction and the likelihood of returning for future visits. The questionnaire that was used was created to measure patient satisfaction issues in general regarding the prophylaxis service using a Likert scale which is a very renowned scale (Taherdoost, 2019). The survey also focused on their intentions about whether they would want to visit dental clinics again or not. The Likert scale is most appropriate because it is clear and simple to use (Batterton & Hale, 2017). This layout encourages patients to respond to a high degree since it is easy to answer and, therefore, a high response rate is consequently achieved (Voutilainen et al., 2016). The Likert scale further assists in a comprehensive assessment of patient sentiment, as it enables various levels of satisfaction, instead of the easy choices of satisfied or dissatisfied (Batterton & Hale, 2017). The ordinal scale also allows us to carry out statistical procedures such as determining the mean or degrees of satisfaction, which is another number that can be computed along with the patient ratings of quality of care or patient outcomes (Gavrilov et al., 2022). The dental prophylaxis services in the case of this device provide room for reporting improvement based on the assessment of patient feedback. In addition, the finding of contributors to patient loyalty and frequent return to business is highly substantial to the success of dental practices.

### 2.4. Statistical Analysis

#### 2.4.1. Data Preparation and Sample Size Power Justification

Data were collected from the integrated information platform that documented both self-evaluations of professionals and patients’ input on their satisfaction. To maintain the study’s validity and integrity, the data were cleaned by dealing with any missing values and inconsistencies. Among the 8067 registered first-time customers, as many as 1500 patients were included in the survey, which is a response rate of 10.2%. Nevertheless, out of the total 1182 answers received, 356 (30.1%) were invalid because of either duplicate or contradictory answers, which indicated a consistency bias. Finally, 826 valid responses were used for the subsequent analyses.

In this study, we assessed the reliability of this two-item questionnaire designed to measure patient satisfaction and intentions to return for dental care, using a sample of 826 respondents. The data were analyzed for internal consistency via Cronbach’s alpha, which is a widely accepted statistical measure for reliability. The overall Cronbach’s alpha for the original items was found to be 0.8902, indicating high internal consistency of the questionnaire. This suggests that both items are measuring a similar underlying construct related to patient experiences in dental care. When excluding each item from the analysis, the Cronbach’s alpha values remained above 0.8023, further confirming that the individual items contribute adequately to the scale’s reliability.

In order to appraise whether the sample recruited was large enough for detecting meaningful differences in patient satisfaction for the standardized dental prophylaxis services, we devised a power analysis before any data was collected (Kang, 2021). A sample size calculation is a critical step in determining the number of subjects needed to attain a given level of statistical power, in turn minimizing the chances of committing a Type I error. The statistical parameters and assumptions were as listed below.

1. Significance Level (α): We fixed the significance level at 0.05, which means 95% of our results are actually true and only 5% are false. This is the way to make sure that the probability of a type I error is limited (wrong hypothesis being rejected) and also the threshold of statistical significance is held at a reasonable level.
2. Statistical Power: The power was set to 0.80, corresponding to an 80% chance of correctly rejecting a false null hypothesis. This is why it is conventionally considered a good research power level for ensuring that the study has the potential to detect the effect, if any.
3. Effect Size (d): Given the absence of prior studies providing an estimate of the effect size within our specific context, we assumed a medium effect size (Cohen’s d = 0.2). A proper effect size, however, is based on Cohen’s criteria. This assumption is based on the common benchmarks used in social sciences to indicate a minimum meaningful difference (Beck, 2013).
4. Standard Deviation (σ): We assumed a standard deviation of 1, which is a conservative estimate and allows for a broad range of variability in the data.

##### Calculation of Sample Size

Using the formula for sample size calculation in two-group comparisons (Chow et al., 2017):

(1) $$n={\left(\frac{Z_{1-\alpha /2}+Z_{1-\beta }}{d/\sigma }\right)}^{2}$$

We substituted our parameters into the formula:

(2) $$n={\left(\frac{1.96+0.84}{0.20/1}\right)}^{2}={\left(\frac{2.80}{0.20}\right)}^{2}={\left(14\right)}^{2}=196$$

Therefore, the required sample size per group would be 196 to detect a 20% effect size with 80% power at a 5% significance level. Hence our final sample of 826 valid replies should be suitable to see an effect size in this situation. Hence, despite the low response rate of 10.2%, our final sample of 826 valid responses was adequate for detecting meaningful differences in patient satisfaction.

#### 2.4.2. Data Analysis

The analysis focused on comparing satisfaction scores and retention rates between the standardized and non-standardized prophylaxis groups. We analyzed the data to determine the impact of the standardized prophylaxis processes service on patient satisfaction and intention to return. The statistical analysis in this study was conducted using SAS software, specifically SAS 9.4. The data underwent a series of analytical procedures to assess the impact of a standardized dental prophylaxis service on patient satisfaction and retention. Initially, the sum scores for each participant were calculated and assessed for normality to ensure the appropriateness of parametric tests.

Demographic features of the study population, like sex, were listed in terms of counts and percentages. Continuous variables, which include the age, were expressed in terms of the minimum and maximum values, means, and the standard deviations (SD). This methodology of descriptive statistics outlines the dispersion of gender and age of participants classified into standardized and non-standardized prophylaxis groups.

Chi-Square was the test selected for sex distribution comparison between the standardized and non-standardized prophylaxis groups because it is the best tool for categorical data analysis. The chi-square test can determine if there are significant differences in proportions between the two groups. This, in turn, makes it very useful in the investigation of the potential disparities in sex distribution.

The Kruskal–Wallis test is a tool for testing the two groups if they have similar age distributions, and it is chosen since it is very useful for non-normally distributed continuous data. This non-parametric method is suitable in cases where the prerequisites of parametric tests, like normality and equal variability among groups, are not satisfied. It is used for age-related data with the possibility of performing a test on the differences in the means of these groups without assuming that the data are normally distributed. This is that case. A *p*-value of less than 0.05 was thought to be statistically significant.

To compare the score changes in terms of patient satisfaction and intention to return between standardized and non-standardized dental prophylaxis groups, we used many statistical methods. The Chi-square test was the main tool to test the significance of the differences in these categorical scores between the two groups. Where sample sizes were small, Fisher’s exact test was used which ensured otherwise correct p-value calculations. In addition, the Kruskal–Wallis H test was carried out to compare the median satisfaction scores without assuming normality checking that the center of the satisfaction groups lies close together.

The employment of the ordinal logistic regression model to investigate patient satisfaction scores and the intention to return, coming from a 5-point Likert scale, can be decided for this reason. The model’s ability to cope with ordinal outcomes is the main reason it is such a good choice for the analysis of Likert data (Wu et al., 2015). The linear regression is based on the assumption of a continuous outcome and might be flawed to the ordinal nature of Likert scales, whereas the ordinal logistic regression gives a more appropriate analytical framework. The latter provides reliable estimates that are not much influenced by outliers and extreme data points, which are the cases where linear regression can give the wrong results (Norris et al., 2006).

## 3. Results

### 3.1. Demographic Analysis

A total of 438 participants were in the standardized group and 388 in the non-standardized group. The sex distribution was almost equal, with 184 females (22.28%) and 204 males (24.70%) in the non-standardized group and 227 females (51.83%) and 211 males (48.17%) in the standardized group. The mean age for the non-standardized group was 38.30 years (SD 11.37), with the minimum being 4 and the maximum age of 75, and for the standardized group it was 38.15 years (SD 12.79 range 8–78 years).

The chi-square test for the sex distribution showed a chi-square value of 1.5961 with 1 degree of freedom and a *p*-value of 0.2065, indicating no statistically significant difference in the distribution of sex between the standardized and non-standardized groups. The analysis of age using the Kruskal–Wallis test yielded a chi-square value of 1.3352 with 1 degree of freedom (df) and a *p*-value of 0.247, suggesting that there is no significant difference in age distribution between the two groups. All the details are presented in Table 1 as follows.

### 3.2. Comparative Analysis

A contingency table analysis was conducted to assess patient satisfaction with dental prophylaxis services among two prophylaxis groups: non-standardized and standardized. The satisfaction ratings were categorized into the following five levels: very poor, unsatisfied, just so-so, satisfied, and absolutely great.

In the non-standardized prophylaxis group, the distribution of satisfaction scores was as follows: 2.58% rated the service as very poor, 23.45% as unsatisfied, 15.46% as just so-so, 54.64% as satisfied, and 3.87% as absolutely great. Comparatively, the standardized prophylaxis group reported slightly higher satisfaction rates with 0.46% rating the service as very poor, 0.46% as unsatisfied, 2.97% as just so-so, 16.67% as satisfied, and 79.45% as absolutely great.

The overall satisfaction scores indicated a higher proportion of patients in the standardized prophylaxis group were either satisfied or absolutely great with the service compared to the non-standardized group. The total number of patients expressing satisfaction across both groups was 43.95% for the highest rating, with a significant difference observed in satisfaction levels between the two groups (Chi-square = 492.8171, df = 4, *p* < 0.0001). Fisher’s exact test also revealed a significant association between the prophylaxis group and patient satisfaction (*p* < 0.0001).

These findings suggest that standardization of the dental prophylaxis process is associated with higher patient satisfaction levels. The standardized group exhibited a markedly lower percentage of patients who were very poor or unsatisfied with the service, indicating a positive impact of standardized procedures on patient experience. The study’s results highlight the importance of standardized prophylaxis procedures in enhancing patient satisfaction. The significant difference in satisfaction scores between the two groups underscores the potential benefits of implementing standardized protocols in dental care settings.All the details are presented in Table 2 as follows.

In the contingency table analyzing patient intentions to return for follow-up visits (Table 3), a stark contrast was observed between the Non-Standardized and Standardized Prophylaxis Groups. The Standardized Group showed a significantly higher proportion of patients with a strong intention to return (79.45% rated as ‘Very likely’), compared to the Non-Standardized Group (63.92% ‘Very likely’). This disparity was statistically significant, as evidenced by a Chi-square test (χ^2^ = 13.7868, df = 4, *p* < 0.0001) and Fisher’s exact test (*p* < 0.0001), indicating that standardization of prophylaxis procedures is associated with increased patient satisfaction and intent to return

The significantly higher retention rates observed in the standardized group highlight the potential benefits of protocol adherence in dental practice. Further research is warranted to elucidate the specific elements of standardized care that contribute to improved patient retention and to assess their impact on long-term oral health outcomes.

### 3.3. Correlation Analysis

In multiple linear regression analysis, the Variance Inflation Factor (VIF) serves as a critical indicator for assessing multicollinearity among predictor variables. High VIF values suggest strong correlations between the independent variables, which may adversely affect the model’s stability and interpretability (Kyriazos & Poga, 2023). In this study, we calculated the linear VIF using the linear model procedure to explain intention to return results in SAS. The computed VIF values were all around 1, which are below 10, indicating the absence of significant multicollinearity issues.

While the linear regression model demonstrated a good fit for explaining the dependent variable of intention to return, it failed to satisfy the fundamental assumption of normality. Analysis of the residuals using a Q–Q plot and statistical tests revealed significant deviations from a normal distribution. Therefore, considering both the characteristics of the intention to return variable and the existing structure of the data, employing an ordinal logistic regression model was deemed a more appropriate next step for analyzing the factors influencing the intention to return variable.

The use of ordinal logistic regression in this study allowed for a more sophisticated analysis that captures the essence of Likert scale data. It provided a comprehensive understanding of the factors influencing patient’s intention to return, respecting the data’s ordinal nature. The model demonstrated a good fit, with an AIC value of 752.691, which is lower than the intercept-only model AIC of 1389.130, indicating a better fit with the inclusion of covariates. The significant chi-square test (χ^2^ = 120.7472, df = 12, *p* < 0.0001) further confirms the model’s overall significance.

The Patient satisfaction score was a significant predictor with a large effect size, as shown by a Wald χ^2^ value of 339.2582 (*p* < 0.0001) and an odds ratio of 24.487, suggesting that a higher satisfaction score is associated with an intention to return. The Group indicator, which differentiates the standardization of the dental prophylaxis service, also showed a significant effect with a Wald χ^2^ value of 86.5029 (*p* < 0.0001) and an odds ratio of 16.063, indicating that standardized dental prophylaxis procedures are positively associated with patient intention to return.

Age was a significant predictor with a Wald χ^2^ value of 13.7749 (*p* = 0.0002) and an odds ratio of 0.969, suggesting that each additional year of age is associated with a slight decrease in the odds of a higher level of return intention. The effect of sex was not significant in this model, with a Wald χ^2^ value of 0.6440 (*p* = 0.4223) and an odds ratio of 0.843, indicating no significant difference in return intention scores between male and female patients. The model’s predictive accuracy was supported by a Somers’ D value of 0.806, indicating a strong association between predicted probabilities and observed responses. The model’s performance is substantiated by the notably high accuracy rate of 89.9% in ordering observations correctly, which validate its consistency and reliability.All the details are presented in Table 4 as follows.

These findings underscore the importance of patient satisfaction levels with dental prophylaxis services, the standardization of dental prophylaxis procedures, and demographic factors in determining patients’ intention to return.

## 4. Discussion

This research constitutes a novel attempt to assess the effects of standardized dental prophylaxis services given to patients in China on patient satisfaction. We confirmed that our survey was reliable enough to measure patient satisfaction based on a rigorous statistical analysis, which was done using SAS. This approach provides a new dimension to understanding patient behavior in this given context.

By comparing the results of two groups, standardized and non-standardized, it was possible to gather the differences in satisfaction scores and intentions to return. In particular, the standardized group achieved higher scores in well-being and were more likely to return. This harmony is in accordance with past studies where patient-centered standardized care can have an impactful influence on patient satisfaction, hence strengthening conclusions made in other fields (Plewnia et al., 2016).

Nonetheless, the distinct demographic and geographic setting of the study provides a new understanding of how these principles specifically fit the Chinese dental care scene. Some results might seem unprecedented, like the marked discrepancies in return intentions among the different groups. This could be explained by the enhanced perception of quality associated with standardized procedures, as patients often equate standardization with reliability and professionalism. Such perceptions may drive their intention to return, even in the absence of extreme disparities in clinical outcomes.

When comparing our results with international studies, it appears that the positive impact of standardized procedures on patient satisfaction is a consistent theme (Khamnil, 2022). However, cultural factors and healthcare structures in various countries may affect the magnitude of this impact. Future comparative studies could provide a broader perspective on how these findings translate across different healthcare systems.

Despite these valuable insights, several limitations must be acknowledged. The cross-sectional nature of the study restricts our ability to establish causality between the standardized service provision and patient satisfaction. Additionally, the specific sample demographics and geographic focus may hinder the generalization of our findings. The study is confined to a single city in China, which limits the ability to extrapolate results to other regions or the broader Chinese population. Factors such as patients’ educational backgrounds and socioeconomic status, which could influence satisfaction and intentions to return, were not explicitly examined, highlighting the need for future research to account for these confounders. Addressing these variables is crucial, as they may significantly impact patient perceptions and experiences in dental care. For example, patients with higher educational levels may have different expectations regarding service quality, while those from varying socioeconomic backgrounds may face accessibility issues that affect their overall satisfaction. By identifying and controlling for these confounders in future studies, researchers can gain a more comprehensive understanding of the factors driving patient satisfaction and retention. Such insights could inform strategies to enhance dental services and ensure they are tailored to meet the diverse needs of patients. Moreover, geographic variations in access to and quality of dental care may affect overall satisfaction rates, yet our analysis did not explore this aspect. Additionally, the reliance on self-reported satisfaction measures introduces potential subjectivity and bias, such as expectation bias, recall bias and social desirability bias (Dowling et al., 2016), which might influence the reliability of participants’ responses and further the interpretation of the findings. The absence of complete electronic medical records for all patients limits our capacity to fully assess the quality of care provided.

Future research should aim to include multiple cities and a larger, more diverse sample to enhance the applicability of the findings. Future research employing qualitative methodologies would be beneficial to explore the specific factors influencing patient satisfaction and retention in prophylaxis services. Such an approach could yield a more nuanced understanding of patient experiences, identifying key elements that contribute to satisfaction and help tailor dental services to meet patient needs effectively.

## 5. Conclusions

This study demonstrates that the implementation of a patient-centered, standardized dental prophylaxis service, underpinned by an integrated information system, has significantly enhanced patient satisfaction and intent to return in a first-tier city in China. These findings not only establish a basis for developing performance evaluation standards and incentive systems for dental prophylaxis practitioners, but also contribute to improved long-term oral health outcomes. The implications of this research provide essential insights for effective dental practice management and highlight the importance of patient-centered approaches in enhancing healthcare delivery.

## Figures and Tables

**Table 1:** Distribution of Sex and Age by Prophylaxis Group.

	Standardized Prophylaxis Group	Non-Standardized Prophylaxis Group
Female	227 (51.83%)	184 (47.42%)
Male	211 (48.17%)	204 (52.5,8%)
Mean(SD)Age	38.15 (12.79)	38.30 (11.37)
(Min.Max) Age	(8.78)	(4.75)
Sex: Chi-square = 1.5961, df = 1, *p* = 0.2065; Fisher’s test *p* = 0.2103		
Age: Kruskal-Wallis: chisq = 1.3352, df = 1, *p* = 0.2479		

**Table 2:** Contingency Table of Prophylaxis Group by Patient Satisfaction Outcome Score.

	Are You Satisfied with This Dental Cleaning Service?				
	1 (Very Poor)	2 (Unsatisfied)	3 (Just So-So)	4 (Satisfied)	5 (Absolutely Great)
Non-Standardized Prophylaxis Group	10 (2.58%)	91 (23.45%)	60 (15.46%)	212 (54.64%)	15 (3.87%)
Standardized Prophylaxis Group	2(0.46%)	2 (0.46%)	13 (2.97%)	73 (16.67%)	348 (79.45%)
Total	12 (1.45%)	93 (11.26%)	73 (8.84%)	285 (34.50%)	363 (43.95%)
Chi-square = 492.8171, df = 4, *p* < 0.0001; Fisher’s test *p* < 0.0001					

**Table 3:** Contingency Table of Prophylaxis Group by Patient Return Intention Results.

	What Is Your Intention to Return for a Follow Up Visit?				
	1 (Very Unlikely)	2 (Unlikely)	3 (Neutral)	4 (Likely)	5 (Very Likely)
Non-Standardized Prophylaxis Group	1 (0.26%)	100 (25.77%)	6 (1.55%)	33 (8.51%)	248 (63.92%)
Standardized Prophylaxis Group	3(0.68%)	1 (0.23%)	2 (0.46%)	82 (18.72%)	350 (79.91%)
Total	4 (0.48%)	101 (12.23%)	8 (0.97%)	115 (13.92%)	598 (72.4%)
Chi-square = 13.7868, df = 4, *p* < 0.0001; Fisher’s test *p* < 0.0001					

**Table 4:** Ordinal logistic regression analyses with patient satisfaction score.

	Odds Ratio	95% CI	*p*-Value
Patients’ satisfaction	24.487	**(17.424, 34.415)**	**<0.001**
Standardized prophylaxis group	16.063	**(8.948, 28.836)**	**<0.001**
Male	0.969	(0.556, 1.279)	0.4223
Age	0.843	**(0.953, 0.985)**	**0.0002**

Significant ORs and *p*-value in bold.

## Data Availability

The data analyzed in this study is subject to the following licenses/restrictions: the data supporting the findings of this study are accessible from the 38 private dental clinics, but access to these data is restricted due to licensing agreements for the current study, and therefore, they are not publicly available. However, interested parties may obtain the data from the authors upon reasonable request, subject to permission. Requests to access these datasets should be directed to taowen86@163.com.

## References

[B1-behavsci-15-00024] Al Ghanem E. J., AlGhanem N. A., AlFaraj Z. S., AlShayib L. Y., AlGhanem D. A., AlQudaihi W. S., AlGhanem S. Z. (2023). Patient Satisfaction With Dental Services. Cureus.

[B2-behavsci-15-00024] Aldahmashi M. A. F., Amaqa Z. I. S. (2022). Oral Hygiene Practices And Teeth Cleaning Techniques: A Comprehensive Review. Journal of Namibian Studies: History Politics Culture.

[B3-behavsci-15-00024] Barranca-Enríquez A., Romo-González T. (2022). Your health is in your mouth: A comprehensive view to promote general wellness. Frontiers in Oral Health.

[B4-behavsci-15-00024] Batterton K. A., Hale K. N. (2017). The Likert scale what it is and how to use it. Phalanx.

[B5-behavsci-15-00024] Beck T. W. (2013). The importance of a priori sample size estimation in strength and conditioning research. The Journal of Strength & Conditioning Research.

[B6-behavsci-15-00024] Bentum-Micah G., Ma Z., Wang W., Atuahene S. A., Bondzie-Micah V. (2020). Perceived service quality, a key to improved patient satisfaction and loyalty in healthcare delivery: The servqual dimension approach. Journal of Health and Medical Sciences.

[B7-behavsci-15-00024] Boparai J. K., Singh S., Kathuria P. (2018). How to design and validate a questionnaire: A guide. Current Clinical Pharmacology.

[B17-behavsci-15-00024] Chen H. (2022). The impact of perceived service quality on patient satisfaction and behavioral intention: The case of a private dental hospital in China. Ph.D thesis.

[B8-behavsci-15-00024] Chow S. C., Shao J., Wang H., Lokhnygina Y. (2017). Sample size calculations in clinical research.

[B9-behavsci-15-00024] Cristache C. M., Mihut T., Burlacu-Vatamanu O. E., Sgiea E. D. (2024). Exploring the Ethical and Legal Aspects of Digital Innovations in Preventive Dentistry. Leveraging digital technology for preventive dentistry.

[B10-behavsci-15-00024] Donnelly L. R., Clarke L. H., Phinney A., MacEntee M. I. (2016). The impact of oral health on body image and social interactions among elders in long-term care. Gerodontology.

[B11-behavsci-15-00024] Dowling N. M., Bolt D. M., Deng S., Li C. (2016). Measurement and control of bias in patient reported outcomes using multidimensional item response theory. BMC Medical Research Methodology.

[B12-behavsci-15-00024] Gao X., Ding M., Xu M., Wu H., Zhang C., Wang X., Si Y. (2020). Utilization of dental services and associated factors among preschool children in China. BMC Oral Health.

[B13-behavsci-15-00024] Gavrilov S. G., Grishenkova A. S., Mishakina N. Y., Krasavin G. V. (2022). Use of a novel Likert scale instrument to assess patient satisfaction following endovascular and surgical treatment of pelvic venous disorders. Phlebology.

[B14-behavsci-15-00024] Harris R., Bridgman C., Ahmad M., Bowes L., Haley R., Saleem S., Taylor S. (2011). Introducing care pathway commissioning to primary dental care: Measuring performance. British Dental Journal.

[B15-behavsci-15-00024] Hescot P. (2017). The new definition of oral health and relationship between oral health and quality of life. The Chinese Journal of Dental Research.

[B18-behavsci-15-00024] Kang H. (2021). Sample size determination and power analysis using the G* Power software. Journal of Educational Evaluation for Health Professions.

[B19-behavsci-15-00024] Khamnil Y. (2022). Development of patient-centered care scales of dentists in primary health care of Thailand: A multigroup analysis. Ph.D thesis.

[B20-behavsci-15-00024] Khan P. (2019). Patient satisfaction with dental services provided by dental students.

[B21-behavsci-15-00024] Kyriazos T., Poga M. (2023). Dealing with multicollinearity in factor analysis: The problem, detections, and solutions. Open Journal of Statistics.

[B22-behavsci-15-00024] Liu J., Zhang S. S., Zheng S. G., Xu T., Si Y. (2016). Oral health status and oral health care model in China. The Chinese Journal of Dental Research.

[B23-behavsci-15-00024] Martignon S., Pitts N. B., Goffin G., Mazevet M., Douglas G. V., Newton J. T., Santamaria R. M. (2019). CariesCare practice guide: Consensus on evidence into practice. British Dental Journal.

[B24-behavsci-15-00024] Minihan-Anderson K. (2023). Ethics and law in dental hygiene-e-book: Ethics and law in dental hygiene-e-book.

[B25-behavsci-15-00024] Naseem S., Fatima S. H., Ghazanfar H., Haq S., Khan N. A., Mehmood M., Ghazanfar A. (2017). Oral hygiene practices and teeth cleaning techniques among medical students. Cureus.

[B26-behavsci-15-00024] Newton J. T., Asimakopoulou K. (2017). Minimally invasive dentistry: Enhancing oral health related behaviour through behaviour change techniques. British Dental Journal.

[B27-behavsci-15-00024] Niranjan V. R., Kathuria V., Venkatraman J., Salve A. (2017). Oral Health Promotion: Evidence and Strategies. Insights into various aspects of oral health.

[B28-behavsci-15-00024] Norris C. M., Ghali W. A., Saunders L. D., Brant R., Galbraith D., Faris P., Approach Investigators (2006). Ordinal regression model and the linear regression model were superior to the logistic regression models. Journal of Clinical Epidemiology.

[B29-behavsci-15-00024] Ou Y., Jing B. Q., Guo F. F., Zhao L., Xie Q., Fang Y. L., Zhou W. (2014). Audits of the quality of perioperative antibiotic prophylaxis in Shandong Province, China, 2006 to 2011. American Journal of Infection Control.

[B30-behavsci-15-00024] Peres M. A., Macpherson L. M., Weyant R. J., Daly B., Venturelli R., Mathur M. R., Watt R. G. (2019). Oral diseases: A global public health challenge. The Lancet.

[B31-behavsci-15-00024] Plewnia A., Bengel J., Körner M. (2016). Patient-centeredness and its impact on patient satisfaction and treatment outcomes in medical rehabilitation. Patient Education and Counseling.

[B32-behavsci-15-00024] Sbaraini A., Carter S. M., Evans R. W., Blinkhorn A. (2012). Experiences of dental care: What do patients value?. BMC Health Services Research.

[B33-behavsci-15-00024] Sedghi L., DiMassa V., Harrington A., Lynch S. V., Kapila Y. L. (2021). The oral microbiome: Role of key organisms and complex networks in oral health and disease. Periodontology 2000.

[B34-behavsci-15-00024] Spanemberg J. C., Cardoso J. A., Slob E. M. G. B., López-López J. (2019). Quality of life related to oral health and its impact in adults. Journal of Stomatology, Oral and Maxillofacial Surgery.

[B35-behavsci-15-00024] Taherdoost H. (2019). What is the best response scale for survey and questionnaire design; review of different lengths of rating scale/attitude scale/Likert scale. International Journal of Academic Research in Management.

[B36-behavsci-15-00024] Voutilainen A., Pitkäaho T., Kvist T., Vehviläinen-Julkunen K. (2016). How to ask about patient satisfaction? The visual analogue scale is less vulnerable to confounding factors and ceiling effect than a symmetric Likert scale. Journal of Advanced Nursing.

[B37-behavsci-15-00024] Wiegand A., Schlueter N. (2014). The role of oral hygiene: Does toothbrushing harm?. Erosive Tooth Wear.

[B38-behavsci-15-00024] Wu W., Jia F., Enders C. (2015). A comparison of imputation strategies for ordinal missing data on Likert scale variables. Multivariate Behavioral Research.

[B39-behavsci-15-00024] Ye H., Meng J., Sun J., Li R., Wei W., Zhang S., Sun Y. (2024). Knowledge, attitude, and practice of dental patients toward dental defects and dental fillings in Jinan, Shandong Province, China: A mediation analysis. BMC Public Health.

[B40-behavsci-15-00024] Zhang X., Liu B., Lynn H. S., Chen K., Dai H. (2022). Poor oral health and risks of total and site-specific cancers in China: A prospective cohort study of 0.5 million adults. EClinicalMedicine.

[B16-behavsci-15-00024] Zhong H. (2019). Growth and transformation of private dental clinics in China: A case study of deron dental. Ph.D thesis.

